# Data-driven diagnosis of spinal abnormalities using feature selection and machine learning algorithms

**DOI:** 10.1371/journal.pone.0228422

**Published:** 2020-02-06

**Authors:** Md. Raihan-Al-Masud, M. Rubaiyat Hossain Mondal

**Affiliations:** Institute of Information and Communication Technology, Bangladesh University of Engineering and Technology, Dhaka, Bangladesh; Universidad de Zaragoza, SPAIN

## Abstract

This paper focuses on the application of machine learning algorithms for predicting spinal abnormalities. As a data preprocessing step, univariate feature selection as a filter based feature selection, and principal component analysis (PCA) as a feature extraction algorithm are considered. A number of machine learning approaches namely *support vector machine* (SVM), *logistic regression* (LR), bagging ensemble methods are considered for the diagnosis of spinal abnormality. The SVM, LR, bagging SVM and bagging LR models are applied on a dataset of 310 samples publicly available in Kaggle repository. The performance of classification of abnormal and normal spinal patients is evaluated in terms of a number of factors including training and testing accuracy, recall, and miss rate. The classifier models are also evaluated by optimizing the kernel parameters, and by using the results of receiver operating characteristic (ROC) and precision-recall curves. Results indicate that when 78% data are used for training, the observed training accuracies for SVM, LR, bagging SVM and bagging LR are 86.30%, 85.47%, 86.72% and 85.06%, respectively. On the other hand, the accuracies for the test dataset for SVM, LR, bagging SVM and bagging LR are the same being 86.96%. However, bagging SVM is the most attractive as it has a higher recall value and a lower miss rate compared to others. Hence, bagging SVM is suitable for the classification of spinal patients when applied on the most five important features of spinal samples.

## Introduction

**Fig 1:**
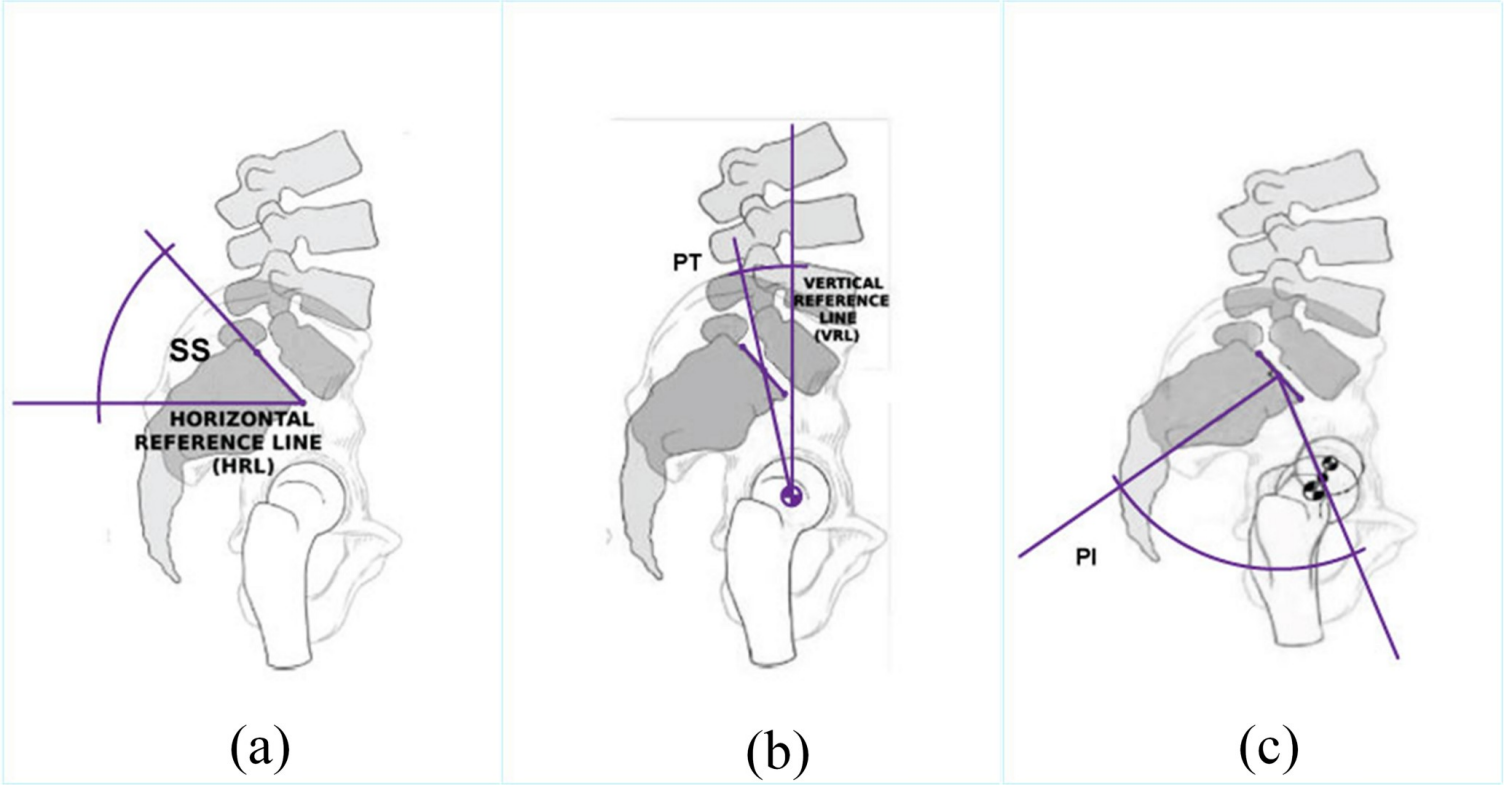
Illustration of a spino-pelvic system (a) pelvic tilt (PT) (b) pelvic incidence (PI) (c) sacral slope (SS) [3].

The spine is the central support structure of human body. The spine connects different parts of human skeleton and keeps the body upright. The spinal cord is often protected by the vertebral column [1–2]. Lumbar vertebrae which is one of the vertebral column segments helps support most of the body weight. The low back is the structure that connects the bones, joints, nerves, ligaments, and muscles which together provide body support, body strength, and body flexibility. Abnormal spinal alignment and posture are generally associated with poor general health, physical function, emotional function, social function, and lower back pain (LBP) [1]. There are a number of attributes for spinal disorder, for example, pelvic tilt, pelvic incidence, sacral slope, etc. Fig 1 illustrates these three attributes of a spino-pelvic system [3].

LBP is often caused by the complications in the lumbar spine [3] affecting the patients' mobility. A minority of cases of LBP can be caused by osteoporosis as well as by trauma to the back. LBP as a form of spinal disorder has a negative socioeconomic impact [4–6,7,8–13]. It is reported in [14] that about 100 million people in the United States has chronic LBP (CLBP). Since spinal disorders in the form of LBP or CLBP cause disability [15], the prevention and early detection of the problems are essential.

A number of research papers report the application of machine learning techniques in medical diagnosis [3, 16–22]. Such techniques enable classification of normal and abnormal cases helping the diagnoses process of patients. For example, machine learning can be used to identify heart disease, cerebral infarction, urological dysfunction, diagnosis of students with learning disabilities, muscle fatigue prediction, etc. [20–21]. In addition, research is going on the aspect of spinal abnormalities and low back pain. Some research papers focus on different classification methods for the referral system known as *clinical decision support system* (CDSS) of LBP patients [14, 18, 22]. On the other hand, some research works identify the degree of importance on lower back pain with abnormal spine whether it is chronic or not [3, 16–17]. The authors in [16] obtain classification accuracy of 85.32% and 79.5% in identifying the abnormal spine using *k-nearest neighbours* (KNN) and random forest algorithms, respectively. In the work [17], both base and meta-level classification algorithms such as naïve Bayes, Bayes net, multilayer perceptron (MLP), random forest, decision table are applied. Prediction accuracy of 81.9%, 83.87% and 83.87% are achieved for random forest, naïve Bayes and MLP classifiers, respectively [17]. In [3], the authors use *kernel with moderate decreasing* (KMOD) and linear kernels of SVM where KMOD kernel exhibits 85.9% classification accuracy. Table 1 briefly summarizes the important works in the research of spinal abnormality.

**Table 1:** Comparison of relevant research works.

Reference	Specific Work on Spinal Abnormality	Machine Learning model used	Dataset
[16]	Identify spinal abnormality	k-NN, random Forest	Kaggle website
[17]	Identify spinal abnormality	naive Bayes- MPL, random forest, etc.	Kaggle website
[18]	Develop a CDSS system	decision tree, random forest, boosted tree	Personal collection of data
[14]	Develop a CDSS system	Classification and regression trees (CART)	Personal collection of data
[3]	Diagnostic of Pathology	SVM with linear and moderate decreasing (KMOD) kernel	supplied by Dr. Henrique da Mota

All the above mentioned research papers only consider the testing accuracy of machine learning based spinal disease prediction. The issues of training accuracy and overfitting are not considered in these papers. Moreover, when searching for the suitable machine learning algorithm, these works do not emphasis on the value of the *miss rate* which is an indication of how many cases of spinal abnormalities are incorrectly detected as normal spine. Therefore, this work focuses on the issues of test and training accuracies, miss rate and recall values in finding the best machine learning algorithm for the prediction of spinal diseases. The contributions of this paper can be summarized as

1. Selecting appropriate attributes of spinal abnormality dataset obtained from the Kaggle repository using *univariate* feature selection method.
2. Extracting features using *principal component analysis* (PCA) based feature extraction method in order to analyse the spinal abnormalities.
3. Applying machine learning algorithms of *support vector machine* (SVM), *logistic regression* (LR) and *bagging* ensemble methods on the important features of spinal abnormalities.
4. Comparing the machine learning algorithms with one another and with the algorithms mentioned in the literature in terms of several factors including train and testing accuracy, recall and miss rate.

The findings may be used as initial steps towards an automatic discrimination between normal and abnormal spines, which may assist practitioners in the clinical treatment of spinal abnormality. The rest of the paper is organized as follows. Section 2 provides a brief description of the overall methodology of this research. Section 3 describes the univariate feature selection and PCA processes for spinal abnormality detection. The SVM, LR and bagging methods are discussed in Section 4. The performance metrics are reported in Section 5. Comparative results on the application of SVM, LR and bagging on the dataset with appropriate features are presented in Section 6. Section 7 provides the concluding remarks.

## Methodology

**Fig 2:**
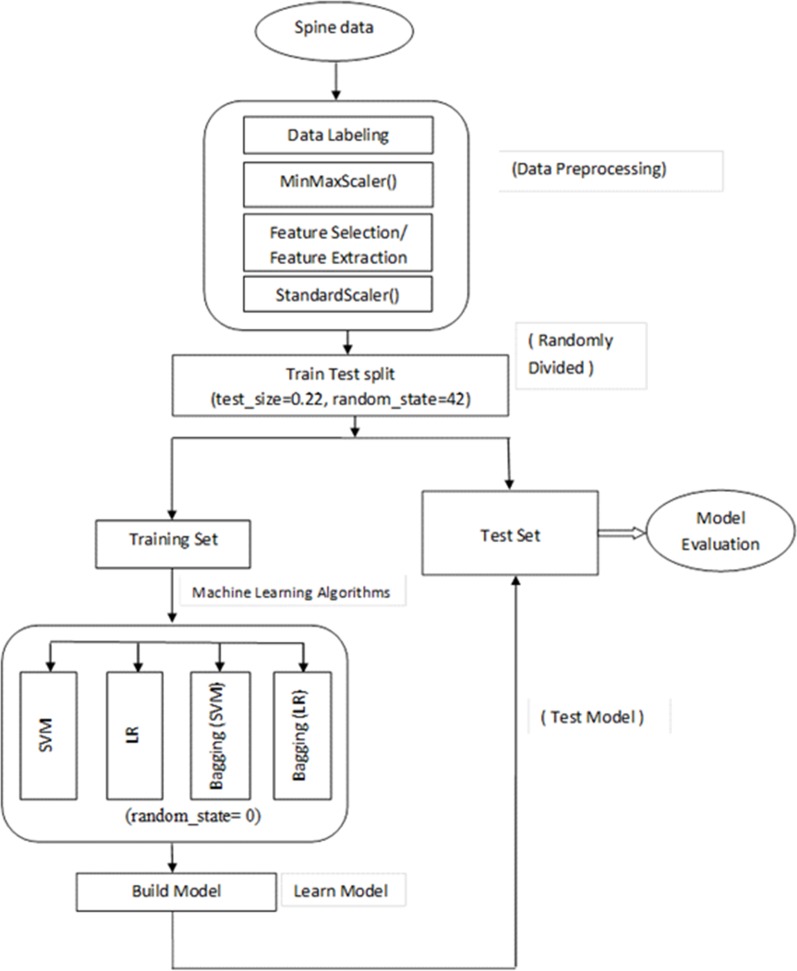
Work flow diagram.

In this research, experiments were performed to classify normal and abnormal spines among the samples available in the dataset. As mentioned earlier, the dataset used in this research was collected from publicly available Kaggle repository [23]. This research work was implemented using scikit-learn which is a machine learning library for the Python programming language. Scikit-learn is built upon NumPy, pandas, and Matplotlib, etc. The work flow diagram of this research is shown in Fig 2. First there were a number of stages for preprocessing of data including data labelling, and data scaling. After that either feature selection or feature extraction were performed. The selected features were then used to classify the data using classification algorithms. This research used categorical data and so SVM and LR were considered as good choices for classification of samples. Furthermore, a form of ensemble classifiers known as *bagging* was also considered in this work. In the experiments with bagging, SVM and LR were used as the base model. We used the *train_test_split()* class from scikit-learn library to split the dataset. This approach of splitting the dataset is known as holdout method. For separating the training and testing data, we also applied the cross validation method using cross_val_score() from scikit-learn library. We trained the model and then test spinal data samples were applied on the trained model to detect abnormal spine.

We found that the dataset had 12 attributes for each of the 310 patients. These records were categorized into two classes, *normal* and *abnormal*. The numbers of normal and abnormal patients were 100 and 210, respectively. As a part of data labelling, the non-numerical categorical attributes were converted into numerical values. The values of abnormal and normal were transformed into 0 and 1, respectively. As a part of data scaling for feature selection, the *min-max scaling* or *min-max normalization* were used as follows:

$$x^{\prime }=\left(x-x_{\min}\right)/\left(x_{\max}-x_{\min}\right)$$ (1)

where *x* is the original value of a feature, *x*_min_ is the minimum of *x*, *x*_max_ is the maximum of *x* and *x*′ is the normalized value of *x*. In order to normalize our data to get the *p* value, the *MinMaxScaler()* function was used. Once the features are selected, the features were normalized using the auto-scaling known as standardization or z-transformation. This was done using the expression below.

$$x^{\prime }=\left(x-x_{\mathrm{mean}}\right)/\sigma$$ (2)

where *σ* is the standard deviation. In order to normalize the selected features to be used by machine learning algorithms, we used *StandardScaler()* from scikit-learn library.

## Feature selection and feature extraction

This section describes feature selection and feature extraction methods used in this research.

### Feature selection

Feature selection is essentially the process of picking some informative and relevant features from a larger collection of features that produce a better characterization of patterns of multiple classes. There are a number of feature selection techniques including filtering based method, wrapper method and embedded methods. As a feature selection method, filter techniques are faster compared to other methods. This is because a filter based method evaluates one feature at a time, rather than evaluating several features together. Furthermore, filter techniques are highly scalable which is important and critical for high-dimensional datasets, relatively simple and efficient, and independent of the underlying classification algorithms [24]. One form of filtering based approach is the *univariate* feature selection method which considers each feature independently that is with regard to the dependent variable. Each feature is scored individually on certain specified criteria and the features are then selected based on the higher scores or higher ranks [25].

In this study, univariate feature selection method was used for identifying the important features of abnormal spine. In order to select the *k* best features from the spinal dataset, we used the *SelectKBest()* class using chi-square distribution function, *chi2()* [25]. The chi-square distribution (chi2) function was used to obtain the *p*-value which is between 0 and 1. The best features were selected by sorting out the features with the lowest *p*-values.

Algorithm 1. Feature selection and other preprocessing

**Input**: A training dataset

**Output**: Selected features ranked according to *p*-value

**Process**:

1: Load the data using *read_csv*() function of panda library

2. Delete any null column using *drop*() function

3. Label the target using *LabelEncoder*() function

4. Divide the data and assign the full dataset (except the target) to *x*

5. Assign the target to *y*

6. Use the holdout method to separate the train and test dataset as x_train, y_train, x_test, y_test using *train_test_split*() function or

apply cross validation using *cross_val_score()*

7. Scale the feature by for a range of 0 to 1 with *MinMaxScaler()* function

8. Obtain selected number of features using *SelectKBest()* function having arguments of *chi2()* and *x_train*

### Feature extraction

One way of feature extraction is the PCA. PCA reduces the dimensionality or the number of features of a large dataset by transforming several features into small number of principal features. The transformed smaller set of variables in PCA still contains the majority of information that is in the actual large dataset. Hence, PCA lowers the complexity of a model and also reduces the chances of overfitting. For the case where the features are measured in different scaling factors, the features need to be standardized as PCA is sensitive to data scaling. Once the features are standardized before PCA, the features are treated with the same importance in the PCA process. In this work, we used the *PCA()* function in in scikit-learn to perform the PCA operation.

## Classification using supervised machine learning

Once the appropriate number of features were selected either by feature selection or feature extraction method, the feature subset was then taken into the classifier training stage where SVM and LR were employed.

### SVM

SVM performs data classification by forming an N-dimensional hyperplane which separates data samples into appropriate classes. SVM can achieve good performance when applied to real world problems [26]. Since SVM is a global representative of the data samples and this reduces the chances of overfitting [27]. In this study, a number of SVM kernels were used: linear, sigmoid, radial basic functions (RBF), and polynomials. [28–29]. These kernels have a parameter *c* which controls the trade-off between obtaining training accuracy and testing accuracy. Furthermore, RBF kernel has a parameter gamma, *γ*, which controls the influence of a single training data sample. The SVM algorithm used in this work is described below.

Algorithm 2. Abnormality detection using SVM.

**Input**: A list of features according to rank

**Output**: Classification Report, Confusion Matrix, Precision-Recall curve

**Process**:

        1. Standardize the selected features using StandardScaler() function

        2. Apply SVM using SVC() function having 'linear' kernel on the selected features

        3. Train the model using selected features

        4. Predict result using test dataset

        5. Evaluate the accuracy of the classifier using *accuracy_score*() function

        6. Use *confusion_matrix*() function to evaluate TP,TN,FP,FN

        7. Use *classification_report*() function to calculate precision, recall and F1-score.

### LR

The LR is a predictive analysis similar to other regression analyses. LR describes the relationship between one dependent binary variable and one/multiple independent variables. In LR, the dependent variable is binary which is contrary to linear regression having continuous dependent variable. LR model has been applied in a number of contexts; which includes: applications to adjust for bias, in comparing two groups in observational studies. LR analysis is part of a category of statistical linear models which consist of fitting a LR model to an observed proportion. The classification process using LR algorithm is described by algorithm 3.

Algorithm 3. Abnormality detection using LR.

**Input**: A list of features according to rank

**Output**: Classification Report, Confusion Matrix, Precision-Recall curve

**Process**:

        1. Standardize the selected features using StandardScaler() function

        2. Apply LR using *LogisticRegression()* function on the selected features

        3. Train the model using selected features

        4. Predict result using test dataset

        5. Evaluate the accuracy of the classifier using *accuracy_score*() function

        6. Use *confusion_matrix*() function to evaluate TP,TN,FP,FN

        7. Use *classification_report*() function to calculate precision, recall and F1-score.

### Bagging

Bagging, also termed as bootstrap aggregating, is essentially the combination of the results of several models. In bagging, samples of observations and subsets of features are created from the actual dataset, with replacement. A base model is created on each subset, where each model is trained independently and in parallel with each other. The combination of the individual models results in the final predictions. The classification process using bagging for the experiments can be described by algorithm 4.

Algorithm 4. Abnormality detection using bagging.

**Input**: A list of features according to rank

**Output**: Classification Report, Confusion Matrix, Precision-Recall curve

**Process**:

Standardize the selected features using StandardScaler() function

Apply bagging using *BaggingClassifier()* function having a parameter *BaseEstimator* on the selected features

Set the *BaseEstimator* to SVC for bagging SVM or

Set the *BaseEstimator* to LogistricRegression for bagging LR

Train the model using selected features

Predict result using test dataset

Evaluate the accuracy of the classifier using *accuracy_score*() function

Use *confusion_matrix*() function to evaluate TP,TN,FP,FN

Use *classification_report*() function to calculate precision, recall and F1-score.

## Performance metric

For the case of biomedical data including spinal samples, total accuracy alone is not sufficient to evaluate a machine learning algorithm. Correct diagnosis of patients is more important. Furthermore, any incorrect prediction of an abnormal patient as a normal patient can be a serious issue. Hence, this work considers a number of metrics for appropriate diagnosis of the patients with spinal abnormalities. For the performance evaluation, true positive (*TP*) refers to the spinal samples correctly classified as abnormal. True negative (*TN*) is the number of normal people who correctly get negative predictions that is they are classified as having normal spinal condition. False negative (*FN*) is the number of undetected patients who actually have spinal abnormality. Furthermore, false positive (*FP*) refers to the number of samples without spinal problem but wrongly classified as abnormal. With this consideration, true positive rate (TPR), true negative rate (TNR), false positive rate (FPR), false negative rate (FNR) are mathematically defined as follows.

$$TPR=\frac{TP}{TP+FN}$$ (3)

$$TNR=\frac{TN}{TN+FP}$$ (4)

$$FPR=\frac{FP}{TN+FP}$$ (5)

$$FNR=\frac{FN}{TP+FN}$$ (6)

The metrics used for performance evaluation are training accuracy, testing accuracy, precision, recall, F1-measure, FNR known as miss rate. In the following these metrics are defined. The accuracy is the percentage of all normal and abnormal vectors that are correctly classified. Accuracy, *ac*, can be expressed as follows.

$$ac=\frac{TP+TN}{TP+TN+FP+FN}$$ (7)

Training accuracy and testing accuracy are defined as the accuracy obtained for training and testing samples, respectively. Precision also known as positive predictive rate is the number of predicted abnormal patients among positive results. Precision, *pr*, can be mathematically written as follows.

$$pr=\frac{TP}{TP+FP}$$ (8)

The term recall refers to the ratio of the number of correctly classified patients with abnormal spine to the total number of patients. Recall indicates the accuracy of a model in predicting the positive class for the case where the actual class is positive. Recall is also known as sensitivity, TPR and detection rate (DR). The term recall, *re*, can be given by

$$re=\frac{TP}{TP+FN}$$ (9)

The F1-Measure, *f*_1_, is the weighted harmonic mean of the precision and recall and represents the overall performance given by

$$f_{1}=\frac{2\times pr\times re}{pr+re}$$ (10)

Confusion matrix is a visualization table that helps to determine the performance of a classification algorithm. Each row and each column in a confusion matrix represent the predicted instances and the actual instances, respectively (vice versa). An important metric is the miss rate, *m*, or FNR given by

$$m=\frac{FN}{FN+TP}$$ (11)

In addition to the aforementioned evaluation criteria, we use receiver operating characteristic (ROC) curve and the area under curve (AUC) to evaluate the pros and cons of the classier. The ROC curve shows the trade-off between the TPR and the FPR. If the ROC curve of a classification algorithm is closer to the upper left corner of the graph or (0,1) coordinate of the ROC space, the classification model is a good one. The precision-recall curves summarize the trade-off between the TPR and the positive predictive values for a predictive model using different probability thresholds. Since the dataset used for this work is imbalance, the precision-recall curves provide a precise view to understand [30].

## Results and discussion

This section provides results and associated discussion on data-driven diagnosis of spinal abnormalities. As mentioned earlier, the results are obtained using scikit-learn library of Python. In the dataset used for the experiments, there were 310 samples where 210 (66.80%) were abnormal and 100 (32.20%) were normal. The experiments were performed considering 241 samples which means 78% of the total samples were training data and 22% were testing data. For the 241 training samples, 161 (66.8%) were abnormal and 80 (33.19%) were normal. For the 69 testing samples, 49 (71%) were abnormal and 20 (29%) were normal. It will be shown later in Section 6.1 that a testing dataset of 22% of the total samples provides excellent fit and better accuracy compared to other proportion of test data. As mentioned earlier, the most important features among the total of 12 attributes were selected using univariate feature selection with the help of *p* value. The lower the value of *p*, the more important the feature was. Table 2 shows the ranking of features based on the univariate feature selection algorithm. It can be seen that *degree spondylolisthesis* has the lowest rank and thus is the most prominent feature.

**Table 2:** Feature name and associated ranking.

Ranked attribute	Attribute name
1	Degree spondylolisthesis
2	Pelvic incidence
3	Pelvic tilt
4	Lumbar lordosis angle
5	Pelvic radius
6	Cervical tilt
7	Sacral slope
8	Direct tilt
9	Pelvic slope
10	Scoliosis slope
11	Thoracic slope
12	Sacrum angle

### Results for SVM and Bagging SVM

This section describes the results for SVM and bagging SVM. In all the experiments of SVM, the linear kernel was used except for the case where all the kernels were compared. Moreover, the value of *random state* variable was set to zero, and probability was set to 'True'. The linear kernel parameter *c* was varied and the best testing accuracy was found at *c* = 0.7and at *c* = 1. The values of parameters *c* and *γ* were varied for RBF kernel to obtain the optimum performance.

Table 3 shows the classification accuracy of SVM obtained for different numbers of features. In this case, the ‘first 2-features’ means the features ranked 1 and 2 from Table 2. It can be seen from Table 3 that for the first 6 features, the highest testing accuracy of 86.96% is obtained and the training accuracy is also close to the highest one being 86.30%. This ensures excellent fit without overfitting or underfitting situations. In this work, the results of linear SVM are shown for *c* = 1. The performance results for SVM using RBF kernel is presented in Table 4. It can be seen that for *c* = 0.9 and *γ* = 1, the best testing accuracy, and the small difference between training and testing accuracies are obtained. The comparison of linear kernel with other kernels is depicted in Table 5. It can be seen from Table 5 that the values for training and testing accuracy for lineal kernel with 6 features are large and very close. This avoids overfitting or underfitting situations. Note that although RBF kernel has the highest training accuracy, its testing accuracy has a much lower value. Hence, for the rest of the paper, linear kernel is considered for SVM.

**Table 3:** Accuracies of SVM for different feature numbers.

Number of features	Testing accuracy (%)	Training accuracy (%)
First 1 feature	82.60	78.00
First 2 features	81.15	77.17
First 3 features	84.05	82.98
First 4 features	79.71	83.40
First 5 features	85.50	86.30
**First 6 features**	**86.96**	**86.30**
First 7 features	86.96	86.30
First 8 features	86.96	86.30
First 9 features	85.50	86.30
First 10 features	85.50	85.47
First 11 features	86.96	85.47
First 12 features	85.50	85.89

**Table 4:** SVM with RBF kernel using 6 features.

*c*–value	Gamma *γ*	Training accuracy (%)	Testing accuracy (%)
0.5	0.1	85.89	81.16
0.7		87.13	81.15
**0.9**		**87.55**	**82.60**
1		87.96	82.60
2		89.21	82.60
5		89.62	81.15
7		90.04	82.60
**0.9**	**0.1**	**87.55**	**82.60**
	0.2	88.38	81.15
	0.3	89.62	81.15
	0.5	90.87	78.26
	0.6	91.28	76.81
	0.9	93.36	76.81

**Table 5:** SVM with different kernels using 6 features.

Different SVM kernels	Training accuracy (%)	Testing accuracy(%)
SVM (kernel = linear, *c* = 1)	**86.30**	**86.96**
SVM (kernel = RBF, *c* = 0.9, *γ* = 0.1)	87.55	82.60
SVM (kernel = poly, *c* = 1, degree = 8)	71.78	71.01
SVM (kernel = sigmoid, *c* = 1)	81.74	82.60

Next, the results for SVM will be evaluated using holdout method for different proportion of test samples. Table 6 presents the training and testing accuracies for the case where 22%, 23%, 24%, 25% and 30% samples are used for testing. Furthermore, both 6 features and all 12 features are taken into consideration.

**Table 6:** Results for different proportion of test samples for SVM.

% of test data	6 features		12 features	
	Training accuracy (%)	Testing accuracy (%)	Training accuracy (%)	Testing accuracy (%)
**22%**	**86.30**	**86.96**	**85.89**	**85.50**
23%	85.29	86.11	85.29	86.11
24%	85.10	86.66	85.10	86.66
25%	84.91	87.17	84.91	87.17
30%	84.79	91.39	84.33	89.24

It can be seen that the best accuracy in classifying spinal abnormality is obtained when 22% samples are used for testing and when 6 features are used instead of all 12. Note that for 22% testing data, the testing accuracy is the highest being 86.96% and the training accuracy is 86.30% which is close to the testing accuracy. The difference between these two accuracy levels is also low indicating excellent fit performance. SVM is also evaluated on the spinal samples after feature extraction using PCA. It is shown in Table 7 that for the case of PAC, the highest testing accuracy is obtained at *n* components of 11 having an accuracy of 85.50%. This value of testing accuracy for PCA is lower than that of feature selection case which is 86.96%. Hence, compared to SVM with feature selection, SVM with PCA method is less effective in classifying spinal data. Next, the performance results for bagging SVM is evaluated.

**Table 7:** Result of SVM-PCA and LR-PCA.

PCA (n components)	SVM-PCA		LR-PCA	
	Training accuracy (%)	Test accuracy (%)	Training accuracy (%)	Test accuracy (%)
1	70.12	72.46	69.71	73.91
2	70.53	72.46	68.87	72.46
3	67.63	76.81	68.87	72.46
4	70.53	75.36	69.29	73.91
5	71.36	75.36	69.29	73.91
6	70.12	73.91	69.70	75.36
7	75.51	71.01	75.10	72.46
8	76.34	66.66	73.02	68.11
9	75.10	72.46	75.93	73.91
10	84.23	75.36	83.40	78.26
**11**	**85.89**	**85.50**	**86.30**	**85.50**
12	85.89	85.50	86.30	85.50

Table 8 shows the training and testing accuracy of bagging SVM for different values of *c*. It can be seen that the best training and testing accuracies are obtained for *c* = 1 and *c* = 7. Table 9 shows the classification accuracies of bagging SVM when applied on different numbers of features of the spinal data. However, it is shown in Table 10 that the precision, recall and F1-score values are better in bagging SVM when *c* = 7. Therefore, for the rest of the paper, bagging SVM with *c* = 7 is considered. It can be seen from Table 9 that for the case of first 6, 7 and 8 features, the values of testing accuracies are the highest, but the differences between the testing and training accuracies are not low. So, this does not result in a good fit of the classifier model. On the other hand, for the case of first 5 features, the testing and training accuracy values are high and close to each other ensuring better fit compared to the case of 6, 7 or 8 features. Hence, for the rest of this work, the first 5 features will be considered for bagging SVM.

**Table 8:** Accuracy of bagging SVM with different values of *c*.

*c* value	Training accuracy (%)	Testing accuracy (%)
0.1	86.72	86.96
0.3	86.72	86.96
0.5	86.72	85.50
0.7	86.30	86.96
0.9	85.89	86.96
1	**86.72**	**86.96**
3	86.30	86.96
5	85.89	86.96
**7**	**86.72**	**86.96**
9	85.89	86.96
10	85.89	86.96
11	85.89	86.96
15	85.47	86.96

**Table 9:** Training and testing accuracies for bagging SVM with different number of features.

No of features	Training Accuracy	Testing Accuracy
First 1 feature	80.49	79.71
First 2 features	79.66	79.71
First 3 features	82.98	84.05
First 4 features	82.98	79.71
First 5 features	86.72	86.96
First 6 features	85.47	88.40
First 7 features	85.06	88.40
First 8 features	85.47	88.40
First 9 features	85.89	85.50
First 10 features	85.06	84.05
First 11 features	85.89	84.05
First 12 features	84.65	84.06

**Table 10:** Elements of confusion matrix for SVM and bagging SVM.

Model	No. of Features	TP	FN	FP	TN	Precision(%)	Recall(%)	F1-score (%)
SVM (*c* = 1)	6	45	4	5	15	90	92	91
	12	45	4	6	14	88	92	90
Bagging SVM (*c* = 1)	5	46	3	6	14	88	94	91
**Bagging SVM (*c* = 7)**	5	47	2	7	13	87	**95.92**	91

**Fig 3:**
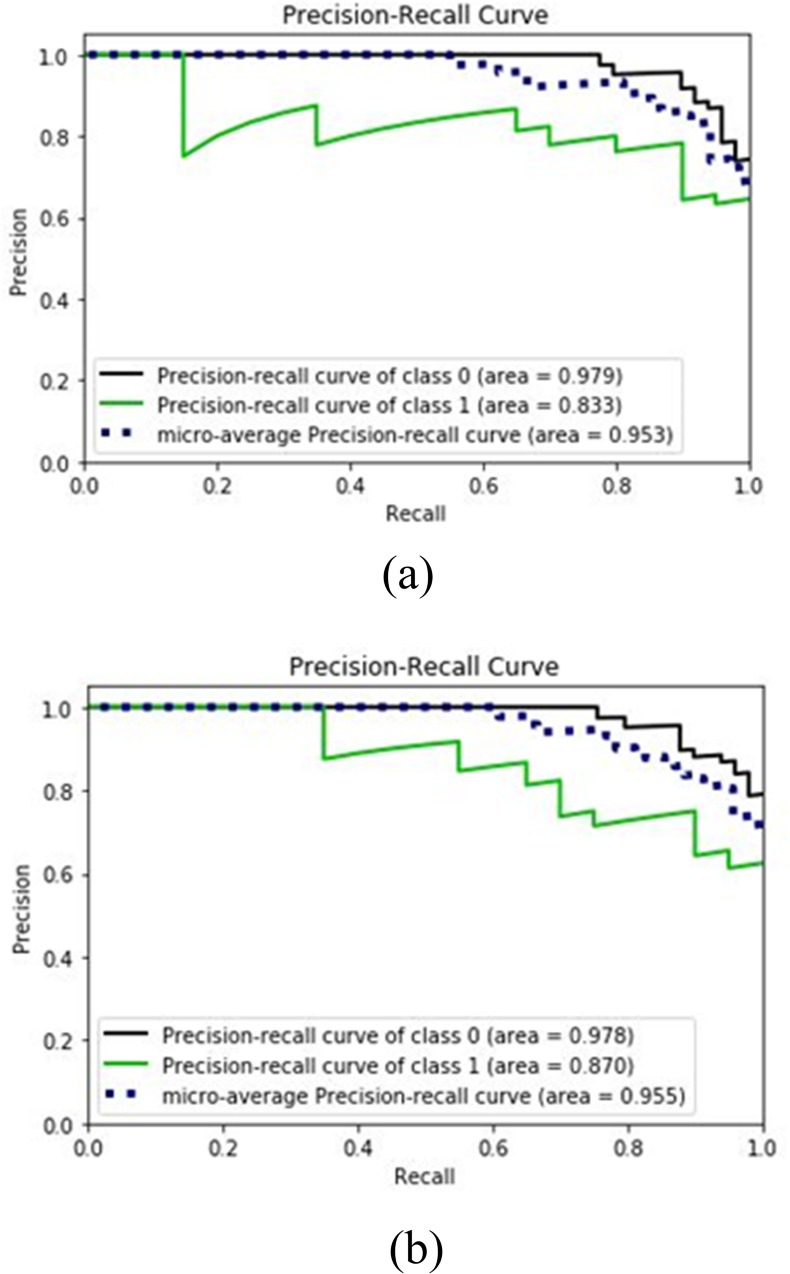
Precision-Recall Curves for (a) SVM with 6 features (b) bagging SVM with 5 features.

In the following section, performance evaluation is done for SVM algorithm while applied on the first 6 features of spinal dataset. Moreover, ROC curves, ROC and precision-recall curves are demonstrated. Table 10 presents the data of confusion matrix elements for SVM with 6 and 12 features, and bagging SVM with 5 features. It can be seen that the TN value for SVM with 6 features is 15, whereas that for 12 features is reduced to 14, the FP values for SVM with 6 features is 5 which is increased to 6 for the case of 12 features. The precision, recall and F1-score for SVM having 6 features are calculated as 90%, 91.84% and 91%, respectively. On the other hand, the precision, recall and F1-score for SVM with 12 features are calculated as 88%, 92% and 90%, respectively. Therefore, for the case of SVM, the values of precision and F1-score are lower for 12 features than 6 features indicating better performance for the case of 6 features. Furthermore, Table 10 shows that bagging SVM exhibits greater recall value of 95.92% at *c* = 7 compared to the value of 94% at *c* = 1. Therefore, *c* = 7 is a better choice for bagging SVM. Fig 3 shows the precision recall plots for abnormal, normal samples and their micro-average for the case of SVM and bagging SVM. Fig 3(A) is for SVM with 6 features, whereas Fig 3(B) is for bagging SVM with 5 features. The micro-average value is higher for bagging SVM is 0.955 which is higher than the value of SVM being 0.953. This indicates the superiority of bagging SVM over SVM for this dataset.

### Results for LR and bagging LR

Next, the performance results for LR and bagging LR will be evaluated. First consider the case where LR is applied on the spinal samples after feature extraction using PCA. Table 7 presents the classification accuracies of LR with PCA. For comparison, values for SVM with PCA are also shown. Table 7 shows that the highest testing accuracy is obtained at *n* components of 11 having an accuracy of 85.50%. Hence, compared to LR with feature selection, LR with PCA method is less effective in classifying spinal data. Hence feature selection method is more suitable and will be considered for the rest of the paper. Table 11 presents the training and testing accuracies of patient classification for the case where 22% patient samples are used for testing. Both LR and bagging LR are considered in Table 11. Furthermore, both 5 features and all 12 features are taken into consideration. It can be seen that the best testing accuracy is obtained in classification of spinal data when 5 features are used instead of all 12. In this case, LR yields training accuracy of 85.47% and testing accuracy of 86.96%, while bagging LR exhibits training accuracy of 85.06% and testing accuracy of 86.96%. The difference between these two accuracy levels is not large indicating excellent fit performance.

**Table 11:** Accuracies for LR and bagging LR.

Classifier	% of test data	5 features		12 features	
		Training accuracy (%)	Testing accuracy(%)	Training accuracy(%)	Testing accuracy(%)
LR	22	85.47	86.96	86.30	85.50
Bagging LR		85.06	86.96	83.82	84.06

Table 12 presents the elements for confusion matrix for samples for LR with 5 features and 12 features and bagging LR with 5 features. From Table 12 it can be seen that the TP value for LR with 5 feature is 45, whereas that for 12 features is reduced to 44; the FN values for 5 features is 4 which is increased to 5 for 12 features. From the values of Table 12, the precision, recall and F1-score can be calculated which are presented later in Table 13.

**Table 12:** Elements of confusion matrix for LR and bagging LR.

Model	No. of Features	TP	FN	FP	TN
LR	5	45	4	5	15
LR	12	44	5	5	15
Bagging LR	5	45	4	5	15

**Table 13:** Comparison of SVM, LR, bagging SVM and bagging LR.

Machine Learning model	Feature selection / extraction	No. of features/dimension	Precision (%)	Recall (%)	Miss rate (%)	F1-score (%)	Testing accuracy (%)	ROC-AUC
SVM	No	12	88.24	91.84	8.16	90	85.50	93.26
	Filter based feature	6	90	91.84	8.16	90.91	86.96	94.08
	PCA	11	88.24	91.84	8.16	90	85.51	
**Bagging SVM (**linear kernel with *c* = 7)	**Filter based feature**	**5**	87.04	**95.92**	**4.08**	91.26	86.96	93.77
LR	No	12	89.80	89.80	10.20	89.80	85.51	93.67
	Filter based feature	5	90	91.84	8.16	90.91	86.96	94.48
	PCA	11	89.80	89.80	10.20	89.80	85.51	
Bagging LR	Filter based feature	5	90	91.84	8.16	90.91	86.96	94.79

**Fig 4:**
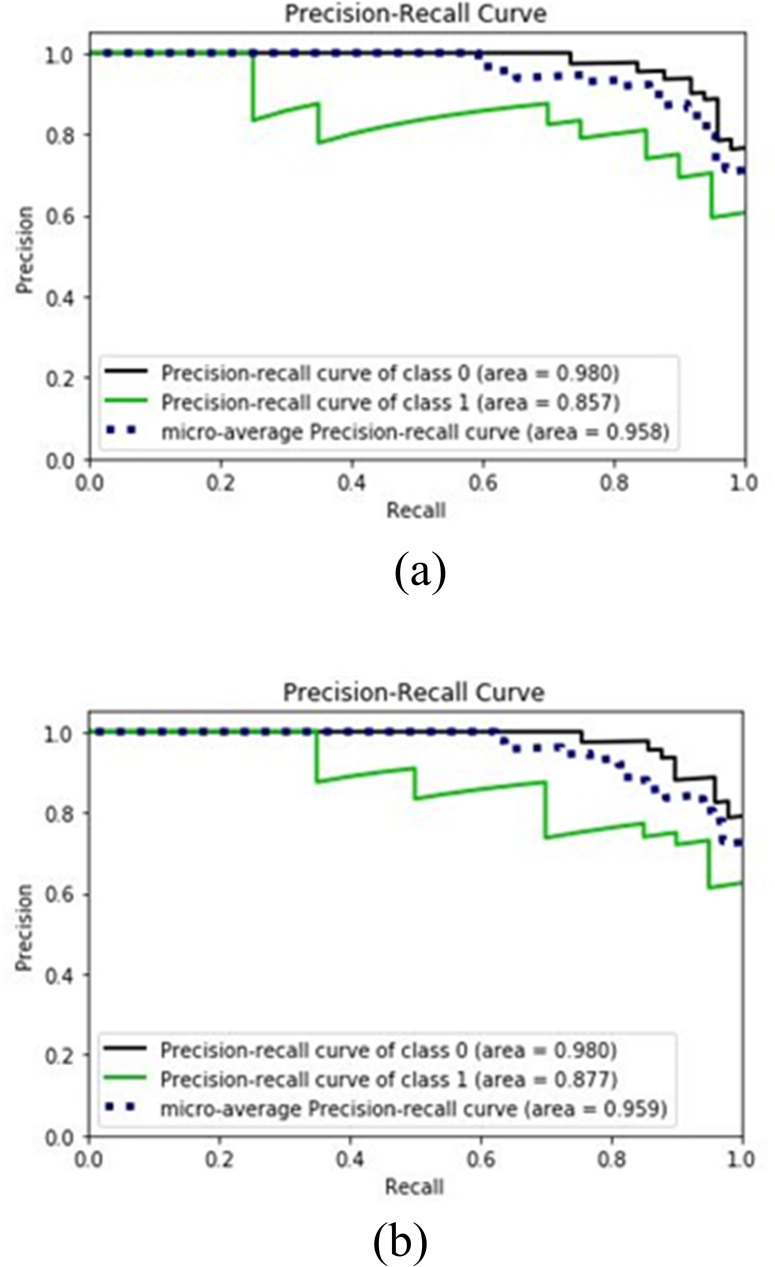
Precision-Recall Curves for (a) LR with 5 features (b) bagging LR with 5 features.

Fig 4 shows the precision recall plots for abnormal, normal samples and their micro average for LR and bagging LR. The micro-average value for bagging LR is 95.9% which is higher than the 95.8% value of LR. This indicates the superiority of bagging LR over LR for this dataset.

### Comparison between SVM, LR and bagging

**Fig 5:**
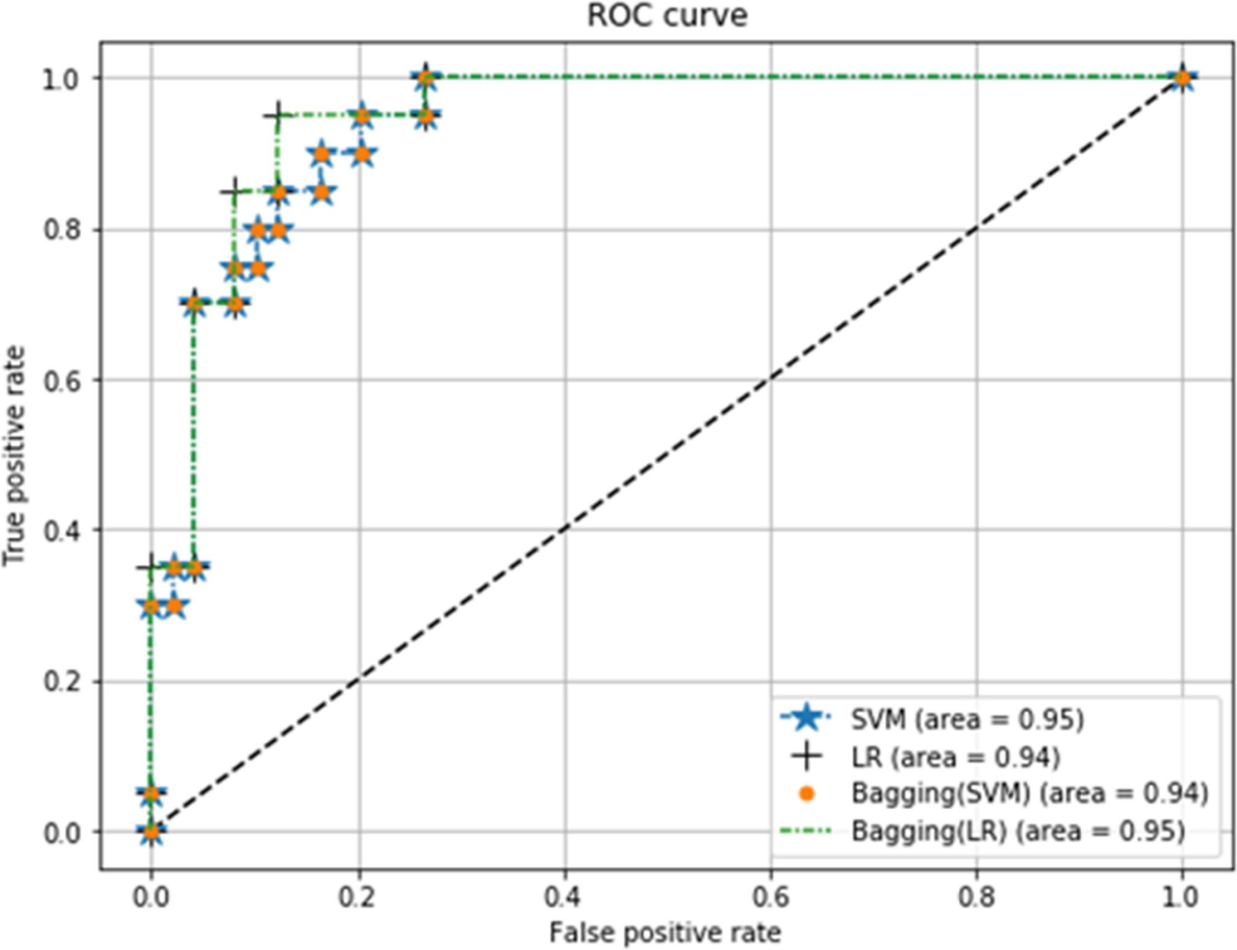
ROC curves with 5 features for SVM, LR, bagging SVM and bagging LR.

This section shows comparative results for SVM, LR, bagging SVM and bagging LR in realizing the classification of spinal data. First of all, we will compare the classifiers using ROC curves. Note that ROC curve is a plot of the FPR (x-axis) versus the TPR (y-axis) for a number of different candidate threshold values between 0 and 1.0. Fig 5 shows the ROC curves for different classifiers. It can be seen that bagging SVM has slightly less AUC value compared to SVM and bagging LR.

Table 13 presents the comparative performance of SVM, LR, bagging SVM and bagging LR in terms of precision, recall and F1-score, miss rate and testing accuracy. Both feature selection and PCA techniques are applied to obtain the attributes to be processed by the classifiers. Results show that for both SVM and LR, feature selection exhibits better classification accuracy than the case of using all features. Moreover, feature selection shows better performance than PCA feature extraction method for classifying the spinal samples. SVM with 6 features and LR with 5 features can more accurately predict spinal abnormality compared to SVM and LR with all 12 features. Furthermore, bagging SVM (*c* = 7) with 5 features has the same testing accuracy (86.96%), but lowest miss rate (4.08%) than other classifiers. In addition, bagging SVM has greater recall value (95.92%) than others at the cost of lower precision value. Hence, bagging SVM having *c* = 7, is the most suitable classifier in predicting whether patients have spinal abnormalities or not.

Table 14 presents the performance results for bagging SVM using cross validation to separate the training and testing samples. For *k* fold cross validation, the value of *k* is varied from 2 to 33. It can be seen that for *k* = 28, the testing accuracy is the highest being 86.94%. This value of accuracy is approximately similar to the testing accuracy of 86.96% obtained with holdout method (22% testing 78% training) shown in Table 13.

**Table 14:** Performance results of bagging SVM using cross validation.

Fold	Precision (%)	Recall (%)	Miss rate (%)	F1-score (%)	Testing Accuracy (%)	ROC_AUC (%)
2	84.70	68.57	31.42	75.78	70.32	82.46
3	85.47	70.00	30.00	76.96	71.71	86.85
4	87.85	75.71	24.29	81.33	76.54	90.41
5	87.32	85.24	14.76	86.27	81.61	90.98
6	88.27	82.38	17.62	85.22	80.71	92.38
7	87.80	85.71	14.29	86.75	82.38	91.64
8	89.16	86.19	13.81	87.65	83.75	93.68
9	89.55	85.71	14.29	87.59	83.76	92.68
10	89.16	86.19	13.81	87.65	83.55	93.00
11	89.42	88.57	11.43	89.00	85.26	90.98
12	88.63	89.05	10.95	88.84	85.13	92.96
13	88.29	86.19	13.81	87.23	83.17	92.53
14	88.78	86.67	13.33	87.71	83.72	92.18
15	89.00	88.57	11.43	88.78	84.94	92.55
16	89.47	89.05	10.95	89.26	85.75	93.08
17	89.76	87.62	12.38	88.67	85.08	94.42
18	88.29	86.19	13.81	87.23	83.26	92.92
19	89.37	88.10	11.90	88.73	85.09	92.95
20	88.52	88.10	11.90	88.31	84.40	93.75
21	89.10	89.52	10.48	89.31	85.53	93.17
22	89.15	90.00	10.00	89.57	86.15	93.15
23	88.63	89.05	10.95	88.84	85.33	92.81
24	89.47	89.05	10.95	89.26	85.73	93.37
25	89.47	89.05	10.95	89.26	85.79	92.69
26	88.89	91.43	8.57	90.14	86.60	92.90
27	88.84	90.95	9.05	89.88	86.34	93.40
28	88.89	91.43	8.57	90.14	86.94	93.59
29	88.79	90.48	9.52	89.62	86.38	93.71
30	89.27	87.14	12.86	88.19	84.70	92.50
31	89.22	86.67	13.33	87.92	84.35	92.60
32	89.27	87.14	12.86	88.19	84.75	93.23
33	88.89	87.62	12.38	88.25	84.66	93.47

Table 15 shows the comparative overview of accuracy of the proposed bagging SVM method with respect to the work reported in the literature. For the proposed bagging SVM algorithm, the results are provided for the case of cross validation and for the case holdout method. It can be seen that the testing accuracy for bagging SVM is almost the same, being 86.94% and 86.96% for cross validation and holdout, respectively. However, the F1-score and recall values are lower for the case of cross validation than holdout. When applied on selected features, the proposed bagging SVM shows a testing accuracy of 86.94% which is better than that reported for k-NN having 85.32% [16], random forest having 81.93% [17], decision table having 81.29% [17] and naive Bayes [17] having 83.87%, SVM linear kernel having 85% [3] and SVM KMOD kernel having 85.9% [3]. Moreover, bagging SVM with or without using cross validation has greater values of precision and recall than values obtained by the algorithms reported in the literature of spinal disorder [16,17, 3].

**Table 15:** Comparison of the proposed work with previous work.

Classifier	No. of features	Accuracy(%)	Recall(%)	F1-score(%)
kNN [16]	6	85.32	90.24	--
random forest [16]	6	79.57	87.50	--
Naïve Bayes [17]	--	77.79	77.7	78.40
random forest [17]	--	81.93	81.9	81.90
Naïve Bayes, MLP [17]	--	83.87	83.9	84.20
random forest, MLP [17]	--	80.00	80.00	80.10
Decision table, random forest [17]	--	81.29	81.30	81.30
Bayes Net, MLP [17]	--	81.93	81.90	82.10
random forest, Naïve Bayes [17]	--	78.38	78.40	79.00
SVM—linear kernel, training size 40% [3]	6	85.00	--	--
SVM -KMOD kernel, training size 40% [3]	6	83.90	--	--
SVM -linear kernel, training size 80% [3]	6	84.30	--	--
SVM -KMOD kernel, training size 80% [3]	6	85.90	--	--
Bagging SVM–linear kernel with *c* = 7, and holdout [Proposed work]	5	86.96	95.92	91.26
Bagging SVM–linear kernel with *c* = 7, and 28 fold cross validation [Proposed work]	5	86.94	91.43	90.14

## 7. Conclusion

This paper diagnoses spinal abnormalities using the concepts of feature selection, feature extraction and machine learning algorithms. For this, investigation is carried by the use of scikit-learn library of Python on a dataset of 310 patients available in Kaggle repository. Using univariate feature selection technique, the degree spondylolisthesis is found to be the most significant attribute of spinal abnormality. A number of machine learning classifiers known as SVM, LR, bagging SVM and bagging LR technique are used to diagnose the spinal abnormality samples. For the SVM algorithm, six features namely degree spondylolisthesis, pelvic incidence, pelvic tilt, lumbar lordosis angle, pelvic radius and cervical tilt are considered. LR, bagging SVM and bagging LR use only five features which are the ones used by SVM except for the cervical tilt feature. In order to obtain the best performance from SVM, the parameter *c* is varied for linear kernel, while the parameters *c* and *γ* are varied for RBF kernel. For the SVM algorithm, the linear kernel is shown to provide better training and testing accuracy than other kernels. Furthermore, for bagging SVM, the parameter *c* of linear kernel is varied to obtain the optimum value. The classifiers perform well when 22% of the spinal abnormality data are used for testing and the remaining 78% as training. Experimental results demonstrate that SVM with 6 features or LR with 5 features have an acceptable recall or detection rate of 91.84% and precision of 90%. The classification accuracies of selected features of spinal samples using SVM, LR, bagging SVM and bagging LR are 86.96%. Precision versus recall, and ROC curves validate the reliability of these classifiers. However, bagging SVM with a parameter value of *c* = 7, has the best miss rate of 4.08% and the best recall value of 95.92% when applied on 5 features for the case of holdout method. When cross validation is used to split the dataset, bagging SVM exhibits a recall value of 91.43% and miss rate of 8.57%. Results indicate that irrespective of cross validation or holdout method, bagging SVM is the most suitable method for data-driven diagnosis of spinal abnormalities compared to other methods described in this paper and in the literature.

One limitation of this research is that the computation time of the classifiers is not studied. Furthermore, several other less popular classifiers have not been investigated in predicting the spinal disorders. As a future work, data may be collected from a larger sample of patients with and without having spinal problem. New classification algorithms can be developed to increase the prediction accuracy of spinal abnormalities.
